# Bovine Follicular Fluid and Extracellular Vesicles Derived from Follicular Fluid Alter the Bovine Oviductal Epithelial Cells Transcriptome

**DOI:** 10.3390/ijms21155365

**Published:** 2020-07-28

**Authors:** Mohammad Mehedi Hasan, Janeli Viil, Freddy Lättekivi, James Ord, Qurat Ul Ain Reshi, Kersti Jääger, Agne Velthut-Meikas, Aneta Andronowska, Ülle Jaakma, Andres Salumets, Alireza Fazeli

**Affiliations:** 1 Department of Pathophysiology, Institute of Biomedicine and Translational Medicine, University of Tartu, Ravila St. 14b, 50411 Tartu, Estonia; mehedi.hasan@ut.ee (M.M.H.); janeli.viil@ut.ee (J.V.); freddy.lattekivi@ut.ee (F.L.); jms.ord18@gmail.com (J.O.); qurat.reshi@ut.ee (Q.U.A.R.); 2 Competence Centre on Health Technologies, Tiigi St. 61b, 50410 Tartu, Estonia; kerstijaager@gmail.com (K.J.); andres.salumets@ccht.ee (A.S.); 3 Department of Chemistry and Biotechnology, School of Science, Tallinn University of Technology, Akadeemia tee 15, 12618 Tallinn, Estonia; agnevelthut@gmail.com; 4 Institute of Animal Reproduction and Food Research, Polish Academy of Sciences, Tuwima St. 10, 10-748 Olsztyn, Poland; a.andronowska@pan.olsztyn.pl; 5 Institute of Veterinary Medicine and Animal Sciences, Estonian University of Life Sciences, Kreutzwaldi 62, 51006 Tartu, Estonia; ylle.jaakma@emu.ee; 6 Department of Obstetrics and Gynecology, Institute of Clinical Medicine, University of Tartu, L. Puusepa St. 8, 50406 Tartu, Estonia; 7 Institute of Genomics, University of Tartu, Riia St. 23b, 51010 Tartu, Estonia; 8 Academic Unit of Reproductive and Developmental Medicine, Department of Oncology and Metabolism, The Medical School, University of Sheffield, Sheffield S10 2SF, UK

**Keywords:** follicular fluid, extracellular vesicles, oviductal epithelial cells, gene expression, fertilization, embryonic development

## Abstract

While follicular fluid (FF) is well known to provide an optimal environment for oogenesis, its functional roles following its release into the oviduct during ovulation are currently elusive. We hypothesized that FF and FF-derived extracellular vesicles (EVs) may be conveyors of signals capable of inducing functionally-relevant transcriptional responses in oviductal cells. The aim of this study was, therefore, to evaluate the effect of FF and FF-derived EVs on the transcriptome of primary bovine oviductal epithelial cells (BOECs). We examined the gene expression of BOECs in three conditions: BOECs cultured with FF, FF-derived EVs, and without supplementations. For each condition, cells were cultured for 6 and 24 h. RNA sequencing results revealed that FF had a stronger effect on BOECs gene expression compared to EVs. We detected 488 and 1998 differentially expressed genes (DEGs) with FF treatment in 6 and 24 h, respectively, whereas only 41 DEGs were detected at 6 h following EV treatment. Pathway analysis of the FF-induced DEGs showed that several pathways were highly enriched, notably oxidative phosphorylation, thermogenesis, arachidonic acid metabolism, and steroid hormone biosynthesis. Some of these pathways have a role in sperm survival, fertilization, and early embryo development. In conclusion, the findings of our study demonstrate for the first time that bovine FF and FF-derived EVs can induce changes in the gene expression of the bovine oviductal cells which, although observed in vitro, may be reflective of in vivo responses which may contribute to a favorable periconceptional microenvironment for sperm survival, fertilization, and early embryo development.

## 1. Introduction

Follicular fluid (FF) is an ovarian fluid that is secreted partly from the follicle and partly as an exudate from the blood plasma [1]. FF plays an essential role in ovarian physiology, steroidogenesis, and oogenesis. It regulates the maturation of the oocyte and its transfer to the oviduct [2]. Several previous studies have shown that some biochemical characteristics of FF play a crucial role in determining the quality of the oocyte as well as affecting fertility and embryonic development in mammals [3,4,5,6]. Several recent studies have shown that in addition to a plethora of metabolic and biochemical substrates, FF contains extracellular vesicles (EVs). EVs are a heterogeneous group of vesicles released from cells with varying sizes and content. Although it is difficult to establish the origin of a given EV and as such the classification of different EV subtypes is constantly evolving [7], three major subtypes have been classified based on their mode of biogenesis: exosomes (typically 40–100 nm), microvesicles (typically 100–500 nm), and apoptotic bodies (typically 500 nm–2 um). Exosomes are produced by the fusion of multivesicular endosomes with the plasma membrane, whereas microvesicles are shed off from the plasma membrane [8]. Cells that are dying release apoptotic bodies, and they appear after the disassembly of an apoptotic cell into fragments [9]. Many studies suggest that these EVs play an essential role in facilitating intercellular communication in many biological systems [10,11,12].

Intercellular communication has a vital role in diverse physiological and pathological processes, including cell proliferation, differentiation, gametogenesis, embryogenesis, and development [13,14]. Many studies have unmasked the existence of EVs in an extracellular environment where they serve as vehicles for the transfer of proteins, lipids, and RNA between cells both locally and remotely [15,16,17]. EVs of FF have been shown to contain miRNAs, and some of these miRNAs target genes that are associated with reproduction, metabolism, and endocrine function, all of which have the utmost importance for a successful pregnancy [11]. It has also been demonstrated that in both in vivo and in vitro conditions, EVs of FF are uptaken by ovarian somatic cells (granulosa cells), and therefore may constitute a new form of ovarian cell-to-cell communication [11,12].

As the site of fertilization and early embryonic development, the oviduct and the oviductal environment play an essential role in embryogenesis [18]. Several studies have shown that the gene expression in the oviduct changes in contact with different factors such as the oocyte, sperm, and embryo, to create an optimal periconceptional microenvironment that supports their survival, fertilization and early embryo development [19,20,21,22]. Although FF, as a carrier of the oocyte, enters the oviduct during ovulation [23], there is limited knowledge of how the oviduct reacts when in contact with FF. We hypothesized that FF or even FF derived EVs induce alterations in the transcriptome of oviductal epithelial cells that might help to create a favorable microenvironment for sperm and oocyte survival, fertilization, and early embryo development.

In this study, we analyzed the transcriptome of the oviductal epithelial cells in contact with FF and FF EVs by employing mRNA sequencing. The differences in the gene expression profiles between supplemented groups and control groups were investigated. As far as the authors are aware, this is the first study to describe the transcriptome of the bovine oviductal epithelial cells in response to bovine FF and FF-derived EVs.

## 2. Results

### 2.1. Size-Exclusion Chromatography Column-Based Isolation of Extracellular Vesicles (EVs) from Bovine Follicular Fluid (FF)

**Figure 1:**
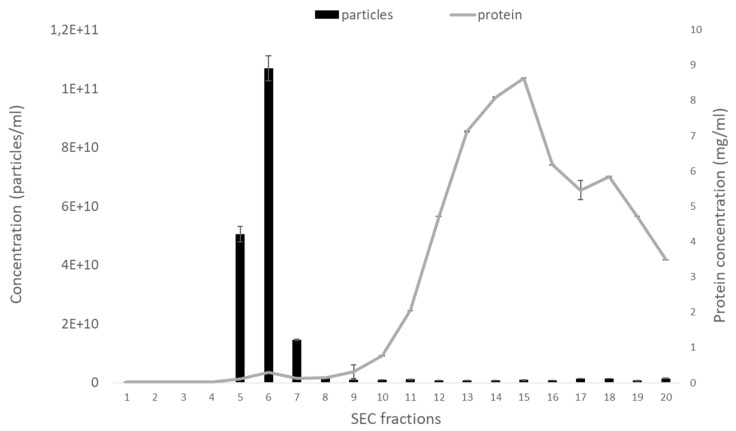
The concentration of extracellular vesicles (EVs) and protein in fractions isolated by size-exclusion chromatography (SEC). Fractions 5–7 contain the highest number of EVs while maintaining relatively low protein contamination. *n* = 3, error bars represent the standard error of the mean (±SEM). EV concentration was analyzed using ZetaView^®^ nanoparticle tracking analyzer (NTA), and the protein concentration was measured using Quick Start™ Bradford Protein Assay.

In order to determine which size-exclusion chromatography (SEC) fractions of bovine follicular fluid contain the highest number of EVs, we collected twenty 500 µL fractions and measured their particle concentration using NTA. We found that fractions 5–7 were highly enriched in EVs (Figure 1), while other fractions either contained no EVs (fractions 1–4) or the concentration of EVs was relatively low (fractions 8–20). By measuring the protein content of each fraction, we found that the protein concentration began to increase after the 10th fraction, whereas fractions 5–7 had minimal protein contamination. Based on these results, in the following experiments, only fractions 5–7 were collected, combined, and used as EV samples.

### 2.2. Characterization of EVs

**Figure 2:**
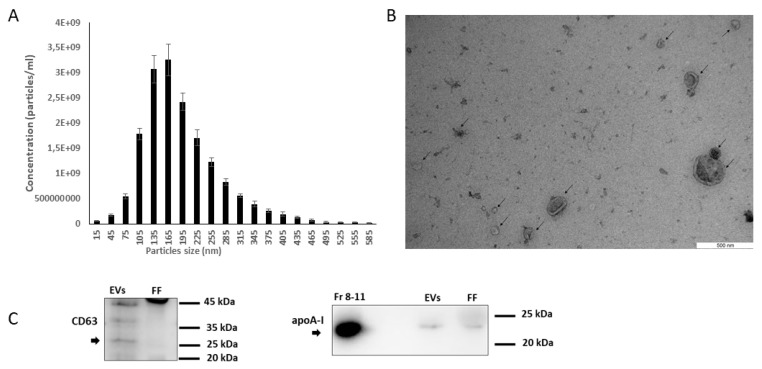
Characterization of follicular fluid derived EVs. (**A**) The size profile of EVs measured by ZetaView^®^ nanoparticle tracking analyzer (NTA), *n* = 3, error bars represent the standard error of the mean (±SEM). (**B**) EVs purified from follicular fluid were analyzed by transmission electron microscopy, where the black arrow points towards the presence of EVs. EVs samples were observed with a JEM 1400 transmission electron microscope at 80 kV, and digital images were acquired with a numeric camera (Morada TEM CCD camera, Olympus, Germany). (**C**) EVs purified from follicular fluid showed a positive signal for EV specific marker CD63 and this particular EV marker was absent in follicular fluid (FF). This comparison showed CD63 as an EV marker was enriched in our samples of EV compared to original FF samples used as control. apoA-I marker was used as a purity control for EVs, and a strong signal of apoA-I was observed in both protein fractions (8–11) and unpurified FF samples compared to the EVs fractions (5–7), which indicates that EVs purified from FF by SEC had little or no contamination. Protein bands were detected using ECL SelectTM Western Blotting Detection Reagent with ImageQuantTM RT ECL Imager.

FF derived EVs were characterized as EVs by NTA, transmission electron microscopy (TEM), and western blot (WB). NTA was performed to determine the concentration and size range of the EVs. Our analysis showed that most of these EVs were under 300 nm in diameter, with a large population in the 50–250 nm range, which is typically considered to be the size range of EVs [24,25] (Figure 2A). TEM analysis confirmed the presence of EVs with their typical cup shape, as indicated by the arrows (Figure 2B). WB analysis showed that the isolated particles expressed EV-specific protein marker CD63, and the apoA-I marker has been used as purity control, as it only was present in protein fractions (8–11), which indicates that the EV fraction isolated from the benchtop column was not contaminated by other non-EV proteins (Figure 2C).

### 2.3. Characterization of BOECs by Immunofluorescence Staining

**Figure 3:**
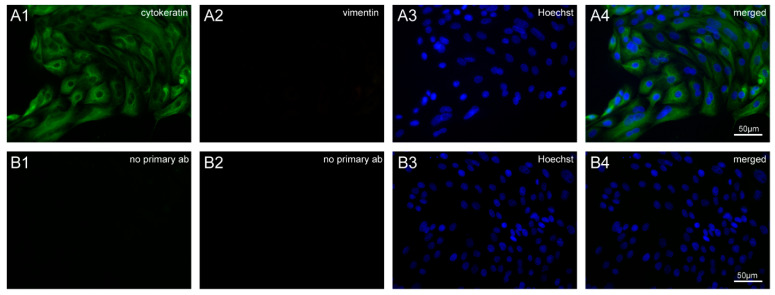
Immunofluorescence staining of isolated bovine oviductal epithelial cells (BOECs). (**A1**) Cells incubated with anti-cytokeratin antibody (green) showing positive staining. (**A2**) No signal was found on the incubation of cells with the anti-vimentin antibody (red). (**A3**) Staining of nuclei with Hoechst (blue). (**A4**) Overlay. (**B1**,**B2**) Negative control staining without primary antibodies. (**B3**) Hoechst staining and (**B4**) overlay of control cells. Images were captured with a Leica DM5500 B microscope equipped with Leica DFC310 camera and processed with ImageJ.

Cytokeratins are present in abundance in epithelial cells, whereas vimentin is mostly found in fibroblasts [26]. This property of cells was exploited using antibodies against cytokeratin and vimentin in order to check for the presence of epithelial or fibroblast cells in our primary cell culture. A strong positive signal was observed with anti-cytokeratin antibody, whereas no signal was observed when cells were incubated with anti-vimentin antibody, which confirmed the presence of epithelial cells only in BOEC culture (Figure 3).

### 2.4. Differential Gene Expression Analysis

The sequencing of the mRNA libraries yielded 4.9 ± 1.4 million reads (mean ± SD) per sample. After the quality control step, 99.0 ± 0.1% of the reads remained in the analysis and were aligned to the *B. taurus* genome assembly (ARS-UCD1.2), out of which 94.4 ± 1.9% aligned to the genome at least once. Read counts were summarized at the gene level, and after removing genes considered not to be expressed in any of the experimental groups, 9678 genes remained in the analysis and were subjected to differential expression testing.

**Figure 4:**
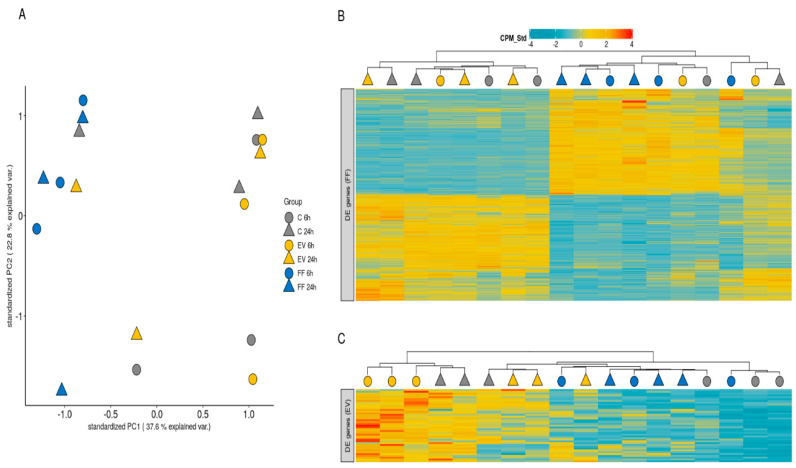
(**A**) Principal Component Analysis (PCA) of standardized (z-score) counts per million (CPM) values of all the expressed genes in the cultured bovine oviductal epithelial cells (BOECs) under control conditions and following supplementation with follicular fluid (FF) or follicular fluid-derived extracellular vesicles (EV). Samples were collected either 6 h or 24 h after supplementation. (**B**) Heatmap of standardized (z-score) CPM values of genes that were differentially expressed either 6 h or 24 h following FF supplementation and hierarchical clustering of samples based on these values. (**C**) Heatmap of standardized (z-score) CPM values of genes that were differentially expressed either 6 h or 24 h following FF EVs supplementation and hierarchical clustering of samples based on these values.

The gene expression profile of the 9678 genes considered to be expressed in the BOECs was considerably variable in the control and EV-supplemented groups, but less so in the FF-supplemented BOEC samples, suggesting a comparatively uniform effect of FF supplementation (Figure 4A). FF supplementation induced modest changes in gene expression profiles at 6 h (184 upregulated and 304 downregulated genes, full list of differentially expressed genes (DEGs) in Supplementary data 1) and more substantial changes to the gene expression profile of the BOECs at 24 h (Figure 4B), with 1022 upregulated and 976 downregulated genes (full list of DEGs in Supplementary data 2). In contrast, the effect of FF-derived EVs was negligible, with 41 genes upregulated and no genes downregulated at 6 h (Figure 4C, full list of DEGs in Supplementary data 3), while no differentially expressed genes were detected at 24 h. Two of the upregulated genes at 6 h following EV supplementation were also upregulated at 6 h following FF supplementation: *DIDO1* and *LOC507443* (Figure 4B, full list of DE genes in Supplementary data 4).

**Figure 5:**
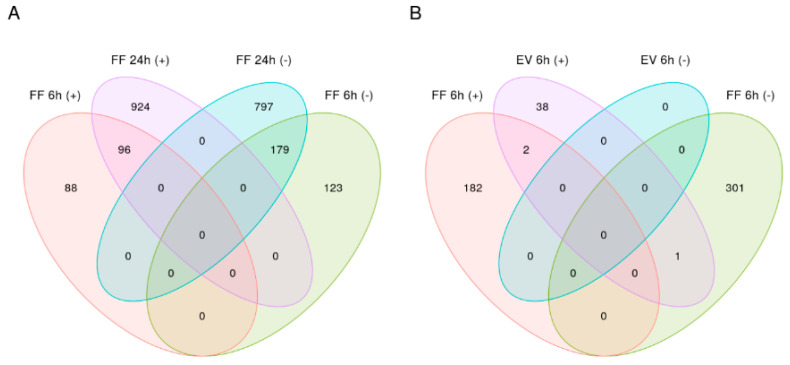
(**A**) Venn diagram representing the number of differentially expressed genes (DEGs), which are common in 6 and 24 h FF treatment. (**B**) Venn diagram representing the number of differentially expressed genes (DEGs), which are common in 6 h FF and 6 h EV treatment.

We detected 96 genes that were upregulated (full list of DEGs in Supplementary data 5) and 179 that were downregulated (full list of DEGs in Supplementary data 6) in response to FF at both time points (Figure 5A). Among these overlapping genes, *NTS* was the most substantially upregulated, and *CYP1A1* was the most substantially downregulated. Interestingly, one gene (*ACKR3*) was commonly differentially expressed in both FF and EV treatment in 6 h time points (Figure 5B). However, with FF treatment, this gene was downregulated, whereas it was upregulated following EVs treatment.

### 2.5. Pathway Enrichment Analysis

Gene Set Enrichment Analysis (GSEA) of the differential expression testing results was conducted based on KEGG pathway annotations for *B. taurus*. In the case of gene expression changes at 6 h after FF supplementation, GSEA yielded two statistically significant (FDR ≤ 0.05) pathways: ribosome (bta03010, normalized enrichment score (NES) = −1.86 and FDR 0.039) and ribosome biogenesis (bta03008, normalized enrichment score (NES) = 1.93 and FDR 0.039). FDR ≤ 0.1 pathways notably included arachidonic acid metabolism (bta00590, normalized enrichment score (NES) = −1.82 and FDR 0.097) and ovarian steroidogenesis (bta04913 normalized enrichment score (NES) = −1.79 and FDR 0.097) that were both enriched with downregulated genes in FF-supplemented BOEC samples (Supplementary Table S1). With the gene expression changes at 24 h after FF supplementation, GSEA resulted in 50 statistically significant (FDR ≤ 0.05) pathways (Supplementary Table S2). Notably, oxidative phosphorylation (bta00190, normalized enrichment score (NES) = 1.78, FDR = 0.010) and thermogenesis (bta04714, normalized enrichment score (NES) = 1.90, FDR = 0.009) were enriched with upregulated genes, whereas the ras signaling pathway (bta04014, normalized enrichment score (NES) = −1.83, FDR = 0.009) was enriched with downregulated genes.

GSEA revealed no significantly enriched pathways at 6 h following EV supplementation (Supplementary Table S3). Although not statistically significant, the tight junction pathway (bta04530) was among the topmost enriched pathways after 6 h of EV supplementation and was enriched with upregulated genes (normalized enrichment score (NES) = 1.54, FDR = 0.052). In the case of 24 h supplementation with EVs, GSEA based on differential expression testing results yielded six significantly enriched pathways (Supplementary Table S4). Notably, oxidative phosphorylation (bta00190) was among the topmost enriched pathways at both timepoints, though it was enriched with downregulated genes at 6 h (normalized enrichment score (NES) = −1.80, FDR = 0.08) and, conversely, enriched with upregulated genes at 24 h (normalized enrichment score (NES) = 1.68, FDR = 0.044).

To identify pathways that were over-represented among genes that were differentially expressed at both time points following FF supplementation, pathway over-representation analysis was conducted separately on significantly up-regulated and down-regulated overlapping genes based on KEGG pathway annotations for *B. Taurus.* No significantly over-represented pathways were detected among overlapping upregulated genes, but eight pathways were significantly over-represented among overlapping down-regulated genes (Table 1), including arachidonic acid metabolism (bta00590, FDR = 0.012), steroid hormone biosynthesis (bta00140, FDR = 0.026) and ovarian steroidogenesis (bta04913, FDR = 0.026).

## 3. Discussion

Analysis of the differential gene expression revealed that bovine FF and FF derived EVs are capable of inducing transcriptomic changes in the bovine oviductal epithelial cells. Moreover, these changes are different between FF and FF EVs, where FF had stronger effects compared to FF EVs regarding the number of differentially expressed genes. At the same time, these effects were different regarding the time duration of incubation. We observed that in the case of FF treatment, these effects were more prominent in 24 h compared to 6 h treatment, whereas the opposite was the case for FF EVs treatment, in which DEGs were detected at 6 h but not 24 h. A possible explanation for the stronger effects of FF compared to FF derived EVs could be that the effects of non-EV components of FF (e.g., hormones, metabolites) outweigh the effects of EVs. Interestingly, most of the genes upregulated in response to EVs at 6 h were not induced by FF at 6 h, suggesting that FF EVs do nonetheless exert some distinct influences independent of other components.

It is very well possible that EVs within the FF have a short lifetime, and their effect is visible only at the early stages of coincubation with BOECs. This may explain why a visible effect of EV coculture with BOEC was observed only at 6 h and not later. Further experimentation is required to better understand how EVs control the gene expression in their recipient cells, for instance time kinetic studies involving time points less than 6 h or between 6 to 24 h could possibly clarify if EVs have a short-lived effect on BOECs.

Studies have shown that several factors, such as spermatozoa [19,27], oocytes [22], embryo [20], and the oviductal fluid itself [28], can change the transcriptomic profile of the oviduct. The present study, for the first time, demonstrated that the bovine FF and FF EVs also could alter the gene expression of the BOECs. It can be hypothesized that the alteration of the observed transcriptomic profile of the oviductal epithelial cells may be necessary to create a favorable environment for sperm survival in the oviduct, fertilization, and even early embryonic development and implantation.

GSEA based on bovine KEGG pathway annotations of differentially expressed genes at 24 h after FF treatment showed that among other pathways, the oxidative phosphorylation and thermogenesis pathways were significantly enriched with upregulated genes in the 24 h FF treatment group. Oxidative phosphorylation is necessary for ATP production to maintain the proper functions of the oviductal cells. The energy produced in the oxidative phosphorylation may induce the thermogenesis pathway. Thermogenesis is heat generation, which could initiate the sperm thermotaxis process. Sperm thermotaxis is the process of sperm guidance by which sperm change the swimming direction and follow the temperature gradients, which leads the sperm towards the fertilization site [29,30]. The above findings may indicate that, by inducing these pathways, FF can contribute to spermatozoa reaching the fertilization site.

GSEA also revealed that biosynthesis of amino acids (FF 24 h) and the tight junction pathway (EV 6 h) were enriched with upregulated genes in response to FF and EVs, respectively. After fertilization, the early embryo usually stays 3–4 days in the oviduct [31]. Several studies have shown that during this pre-implantation period, pyruvate, lipids, lactate, and amino acids are the primary source for embryo nutrients [32,33,34]. Therefore, if these pathways are induced by FF or FF derived EVs, they may contribute to early nutrient supplementation for embryo development. Furthermore, it has been reported that during these periods, oviductal epithelial cells transfer nutrients to the early embryo [35], and tight junctions between oviductal epithelial cells play an essential role in this process [36,37].

Among the downregulated pathways in cells with FF treatment, some pathways were over-represented in both time points, notably arachidonic acid metabolism and steroid hormone biosynthesis, which were highly enriched with downregulated genes. Oxygenation of arachidonic acid generates several metabolites capable of modulating immune reactions [38,39]. As a nonself entity within the female reproductive tract, the maternal immune system may register spermatozoa as a pathogen, and thus local modulation of the immune system could be necessary for spermatozoa to reach the fertilization site successfully [40,41].

The proper balance of steroid hormones (estradiol, progesterone) is crucial for oocyte transport, fertilization, and successful pregnancy. Decreased concentration of estradiol is necessary for the transportation of the cumulus-oocyte complex (COC) to the fertilization site from infundibulum. However, the levels of steroid hormones fluctuate before and after fertilization [42,43]. These findings indicate that FF may induce downregulation of steroid hormone biosynthesis, which could, therefore, assist with proper transportation of COC to the fertilization site. One of the previous studies also demonstrates that estradiol from the follicular fluid is capable of altering the expression of genes associated with inflammation and DNA damage response in the oviductal epithelial cells [44].

In addition, some genes were significantly upregulated in the 24 h FF treatment that have known functions in reproduction but which were not represented in any significant pathways. Among them is Prostaglandin E Synthase2 (PTGES2), which is involved in prostaglandin biosynthesis. Prostaglandins have an essential role in oviductal muscular activity [45]. Prostaglandins enhance the contractions of the oviductal smooth muscle cells that help spermatozoa to reach the fertilization site and also aid the transfer of the embryo along the oviduct toward the uterus [45,46,47,48]. Neurotensin (NTS) was the most significantly upregulated gene in both time points with FF treatment. Studies in both mice and Japanese black cattle have shown that NTS is involved in sperm capacitation and acrosome reaction [49,50]. These two events are necessary for sperm to be able to fertilize the egg, which suggests that one of the functions of FF in the oviduct is to enhance fertilization by inducing the upregulation of NTS.

The role of follicular fluid in preparation of preconceptional environment has received comparatively little attention, and therefore our study has addressed a fundamental knowledge gap. However, some limitations of our study must be acknowledged. Expression profiles were considerably variable, probably because of the inherent heterogeneity of primary cells. In addition, because the experiments were carried out over three different days using separately sourced FF and FF derived EVs, the variation may have been driven by temporal influences or inter-individual differences in FF composition. Inter-individual variability between source individuals should be further addressed in future studies by using cell cultures derived from a greater number of individuals. Assays to confirm the uptake of EVs by BOECs should also be considered, as well as validation of differentially expressed genes by qPCR. Finally, although we observed changes in specific pathways and genes that can be interpreted as supportive of reproductive processes, more targeted experiments are required to test these hypotheses in more detail.

In conclusion, the findings of our study demonstrate that bovine FF and FF-derived EVs can induce changes in the gene expression of the bovine oviductal cells which, although observed in vitro, may be reflective of in vivo responses contributing to a favorable periconceptional microenvironment for sperm survival, fertilization, and early embryo development.

## 4. Materials and Methods

### 4.1. Isolation and Culture of Bovine Oviductal Epithelial Cells

Oviducts from three cows (Holstein) with attached ovaries were collected from the slaughterhouse (Rakvere, Estonia) and transported to the laboratory in saline within 4 h after animal slaughter. Oviducts were taken from ovaries that belonged to the stage I of the estrous cycle (bright red *corpus luteum*, no vasculature around the surface *corpus luteum*, 0–4 days post ovulation) [51].

The oviducts were washed three times with saline, trimmed, and cleaned of outer tissues. The oviductal epithelial cells (BOECs) were gently squeezed out of the oviductal tissue with a sterile glass slide, and the cells were collected directly into the washing buffer. The washing buffer consisted of Dulbecco’s phosphate-buffered saline (DPBS, Verviers, Belgium) supplemented with 1% penicillin-streptomycin (P/S, Gibco, Bleiswijk, The Netherlands) and 1% amphotericin B (Sigma-Aldrich, Saint Louis, MO, USA). We only used the ampulla part for BOECs extraction. The BOECs were washed twice with washing buffer and centrifuged at 50 g for 2 min between washes. Finally, BOECs were washed once in washing buffer supplemented with 5% fetal bovine serum (FBS, Gibco^TM^), plated in Dulbecco′s Modified Eagle Medium/Nutrient Mixture F-12 (DMEM-F12, ThermoFisher Scientific Inc., Santa Clara, CA, USA) supplemented with 10% FBS, 1% P/S and 0.01% amphotericin B and cultured in a humidified atmosphere at 38.8 °C with 5% CO_2_. The cells were left to attach for 72 h before changing the medium. BOECs used during experiments were frozen once and passaged twice before being used in the experiments. In brief, cells were washed thoroughly using DPBS and incubated with trypsin at 38.8 °C until the cells detached utterly. After that cells were frozen using freezing media (70% DMEM-F12, 20% FBS and 10% dimethyl sulfoxide (DMSO)).

### 4.2. Collection of Follicular Fluid

The ovaries for FF collection were obtained and transported in the same manner as oviducts. After washing the ovaries thrice in saline, the FF was collected only from large follicles (>9 mm) by a vacuum pump, maintaining a constant pressure of 100 Pa. FF was centrifuged at 300× *g* for 10 min to remove any cells. The supernatant from the previous step was then centrifuged at 2000× *g* for 10 min to remove cell debris. FF was stored at −80 °C for further experiments.

### 4.3. Isolation of EVs from Bovine FF

FF (10 mL) that was used for EV isolation was diluted 1:1 with DPBS to decrease the viscosity and then centrifuged at 300× *g* for 10 min, followed by centrifugation at 2000× *g* for 10 min. In order to remove apoptotic bodies, the sample was centrifuged at 20,000× *g* for 30 min, and then the supernatant was filtrated through a 0.2 µM syringe filter. The sample was concentrated down to 500 µl using Amicon^®^ Ultra-15 centrifugal filter units (10 kDa). EVs were isolated using size exclusion chromatography (SEC) benchtop columns (Econo-pac^®^ Disposable chromatography column, Bio-Rad, Berkeley, CA, USA) filled with cross-linked 4% agarose matrix of 90 µm beads (Sepharose 4 fast flow™, GE HealthCare Bio-Sciences AB, Uppsala, Sweden). Twenty fractions, each 500 µl, were collected, and the protein concentration of each fraction was determined with Bradford assay using Quick Start™ Bradford Protein Assay (Bio-Rad, Berkeley, CA, USA) according to the manufacturer′s protocol. Particle concentration in the fractions was measured using nanoparticle tracking analysis (NTA, ZetaView, Particle Metrix GmbH, Inning am Ammersee, Germany). Based on these analyses, fractions 5–7 (1.5 mL) were pooled together, concentrated with Amicon^®^ Ultra-15 centrifugal filter device (10 kDa cut-off) and were used in further experiments.

### 4.4. Nanoparticle Tracking Analysis

The size and concentration of the EVs in the EV samples were determined with the ZetaView PMX 110 NTA instrument (Particle Metrix GmbH, Inning am Ammersee, Germany). We followed the standard manufacturer′s procedure for nanoparticle size distribution and concentration measurement. A 100 nm particles standard (Applied Microspheres BV, Leusden, The Netherlands. Catalogue no. 10100) was used to calibrate ZetaView^®^ before the measurements. ZetaView measurements, each particle was counted and size distributed at three cycles of 11 frames per cycle were conducted under the following settings: sensitivity 85, shutter speed 70, and frame rate 30 fps. All samples were measured in triplicates. In between measurements, the measurement cell was washed thoroughly using Milli-Q^®^ water and DPBS before the injection of the next sample in order to minimize the inter-sample contamination.

### 4.5. Western Blot Analysis

EV fractions (pooled fractions 5–7) and protein fractions (pooled fractions 8–11) of bovine follicular fluid were purified with SEC as described earlier and the samples were concentrated with Amicon^®^ Ultra-15 centrifugal filter device (10 kDa cut-off) to 300 µl. Proteins were precipitated by adding 100 µL of water, 400 µL of methanol and 100 µL of chloroform to 300 µL of concentrated sample. The solution was mixed and centrifuged 14,000× *g* for 5 min at room temperature. The top layer was carefully removed, the precipitated proteins were washed with 400 µL of methanol and centrifuged again. The pellet was air-dried and resuspended in 0.25% SDS. The follicular fluid sample was prepared in parallel and the protein concentrations were determined by Bradford assay. 30 µg of protein was suspended in non-reducing Laemmli buffer (for CD63 detection) and 50 µg of protein was suspended in reducing Laemmli buffer (for apoA-I detection) and incubated for 5 min at 95 °C. Protein samples were separated by 12% SDS-PAGE gel electrophoresis according to standard protocol. Proteins were transferred onto polyvinylidene difluoride membrane (Thermo Scientific, Rockford, IL, USA) followed by blocking for one hour at room temperature. Blocking buffer for CD63 detection was 5% BSA (Pan Biotech GmbH, Aidenbach, Germany) in PBS-T (PBS + 0.05% Tween−20) and 5% nonfat dry milk in PBS-T for apoA-I detection. Subsequently, the membrane was incubated with the primary anti-CD63 antibody (ab68418, 1:500, Abcam, Cambridge, UK) and anti-apoA-I antibody (sc-376818, 1:1000, Santa Cruz Biotechnology Inc., Dallas, TX) overnight at 4 °C followed by incubation with horseradish peroxidase-conjugated goat anti-rabbit secondary antibody (G21234, 1:20,000, Invitrogen, Thermo Fisher Scientific, Eugene, OR, USA) and goat anti-mouse secondary antibody (G21040, 1:20,000, Invitrogen, Thermo Fisher Scientific, Eugene, OR, USA) for 1 h at room temperature. The membrane was washed three times for 5 min in PBS-T after each incubation step. Protein bands were detected using ECL SelectTM Western Blotting Detection Reagent with ImageQuantTM RT ECL Imager (both GE Healthcare, Buckinghamshire, UK).

### 4.6. Transmission Electron Microscopy

EV suspension was deposited on Formvar-carbon-coated 200 mesh copper grids (Agar Scientific, Stansted, UK). The method described by Thery et al. 2018 [7] was followed for transmission electron microscopy (TEM) analysis. Before contrasted in uranyl oxalate (mixture of 4% uranyl acetate (Polysciences, Warrington, PA, USA) and 0.15 M oxalic acid (Sigma-Aldrich, Schnelldorf, Germany)) and embedded in a mixture of methylcellulose (Sigma-Aldrich, Schnelldorf, Germany) and uranyl acetate (Polysciences, Warrington, PA, USA), EVs were fixed on grids in 2% paraformaldehyde (Sigma-Aldrich, Schnelldorf, Germany) and 1% glutaraldehyde (Polysciences, Warrington, PA, USA). Samples were observed with a JEM 1400 transmission electron microscope (JEOL Ltd. Tokyo, Japan) at 80 kV, and digital images were acquired with a numeric camera (Morada TEM CCD camera, Olympus, Germany).

### 4.7. Immunofluorescence Staining

BOECs grown on glass coverslips were washed and fixed with 4% paraformaldehyde for 10 min at room temperature, followed by additional fixing and permeabilization with cold methanol for 10 min on ice. After washing, the cells were incubated in blocking solution (4% normal goat serum in PBS) for 1 h at room temperature, and then with anti-Cytokeratin (C2562, 1:250, Sigma-Aldrich) and anti-Vimentin (PLA0199, 1:250, Sigma-Aldrich, USA) antibodies in blocking solution for 1 h at room temperature. Negative control cells were incubated in a blocking solution without primary antibodies. Next, cells were washed three times with PBS and incubated with Alexa Fluor 488 goat anti-mouse and Alexa Fluor 594 goat anti-rabbit secondary antibodies (A11029 and A11012, respectively, 1:500, Invitrogen, Thermo Fisher Scientific, Eugene, OR, USA) in blocking solution for 45 min at room temperature in the dark. The nuclei were counterstained with Hoechst 33,342 (Thermo Fisher Scientific), and the coverslips were mounted with Fluorescence Mounting Medium (Dako Glostrup, Denmark). Images were captured with a Leica DM5500 B microscope equipped with Leica DFC310 camera (Leica, Wetzlar, Germany) and processed with ImageJ [52].

### 4.8. Supplementation and Bovine Oviductal Epithelial Cells Culture

BOECs were cultured in 12 well plates until cells attained 80% confluency. The BOECs were washed twice with pure (without any FBS or antibiotics) DMEM-F12 media, and cells were treated as follows: (1) supplemented with FF (200 µl/mL) (2) supplemented with FF-derived EVs (3.0 × 10^6^/µl, based on our preliminary results this concentration corresponds to physiological EV concentration in large FF) and (3) untreated (control group). BOECs were collected for further experiments in 6 and 24 h after treatment. As FBS contains EVs [53], all media used in supplementation experiments were EV depleted by using Amicon^®^ Ultra-15 Centrifugal Filter Units (100 kDa). We conducted the same experiments on three different days, along with collecting follicular fluid from different sources, but on each day we used FF and FF EVs from the same pool to carry out the supplementation.

### 4.9. RNA Isolation, Library Preparation, and Sequencing

#### 4.9.1. RNA Isolation

After the respective incubation periods, the media was discarded, and total RNA was extracted from BOECs with the TRIzol^®^ reagent (TRIzol^®^ reagent; Invitrogen, Thermo Fisher Scientific, Eugene, OR, USA) according to the manufacturer′s protocol. In order to increase the yield of RNA, 2 µL of GlycoBlue (GlycoBlue™ Coprecipitant, Thermo Fisher Scientific, Bleiswijk, The Netherlands) was added to the lysis buffer. The RNA pellet was washed three times, with 75% ethanol. Quantity of RNA was measured using Qubit™ RNA HS Assay Kit (Q32852, Thermo Fisher Scientific), and the quality of extracted RNA was determined by the Bioanalyzer Automated Electrophoresis instrument (Agilent Technologies, Santa Clara, CA, USA) using Agilent RNA 6000 Pico Kit (Agilent Technologies).

#### 4.9.2. RNA-Seq Library Preparation

RNA sequencing libraries were generated by using Smart-seq2 methodology [54] with slight modifications. Twenty ng of total RNA was used for cDNA synthesis and ten cycles of PCR for pre-amplification, instead of using single cells as supposed by the original Smart-seq2 protocol. KAPA HiFi DNA polymerase was replaced with Phusion High-Fidelity DNA Polymerase (Thermo Scientific) compatible with the original protocol. Two μL of diluted cDNA was applied to the dual-index library preparation using Illumina Nextera XT DNA Sample Preparation Kit (FC-131-1024). To clean-up all steps and for size selection 200–700 bp, we used AMPure XP beads (Beckman Coulter). All samples were pooled into a single library by equal concentration and sequenced on Illumina NextSeq500 using High Output Flow Cell v2.5 (single-end, 75 bp read length).

#### 4.9.3. Processing, Alignment, and Quantification of RNA-Seq Reads

The quality of raw reads was assessed using FASTQC v0.11.8 [55]. Trimmomatic v0.39 was used for read trimming and removal of adaptor sequences using the following parameters: LEADING:20, SLIDINGWINDOW:4:15, ILLUMINACLIP: *:1:30:15 and MINLEN:25.

Reads were aligned to the *Bos taurus* genome assembly (ARS-UCD1.2) obtained from the Ensembl database [56]. The alignment was performed using HISAT2 [57]. with default parameters and with the inclusion of splice site information derived from the corresponding Ensembl *B. taurus* annotation file (ARS-UCD version 1.2.97). Gene-level read counts were obtained using featureCounts [58]. with default parameters, using the Ensembl *B. taurus* annotation file (ARS-UCD version 1.2.97) for genomic feature annotations. Genes with at least 10 counts for all the samples in at least one of the experimental groups were retained in the analysis.

### 4.10. Differential Gene Expression Analysis

Differential expression analysis was carried out in R version 3.6.1 using the edgeR package version 3.26.8 [59]. Tagwise dispersion estimates were obtained based on the trended dispersions, and statistical comparisons were performed using a generalized linear model followed by likelihood ratio tests, also accounting for the experiment batch. We considered the differential expression of genes with a false discovery rate (FDR) ≤ 0.05 to be statistically significant.

Gene set enrichment analysis (GSEA), and pathway over-representation analysis was conducted using the clusterProfiler package [60] and KEGG Pathway database annotations. The GSEA method was used for full gene lists, including both statistically significant and nonsignificant results, obtained from differential expression analysis that were ranked by *−log_10_p* × *log_2_FC*, where *p* denotes unadjusted *p*-values and FC the fold-change. As GSEA benefits from utilizing full-length unfiltered expression datasets [61], it can be used in cases where preceding differential expression analysis does not result in any individually statistically significant genes. The genes can still be ranked using the aforementioned metric and GSEA can potentially uncover significant underlying patterns (pathway annotations) in the ranked gene list. Pathway over-representation analysis was used in the case of intersected gene lists and conducted separately for up and downregulated gene sets. Results obtained from pathway analyses were considered to be statistically significant at FDR ≤ 0.05.

Principal components were calculated using prcomp function from the Stats package and visualized using the ggplot2 package (https://cran.r-project.org/web/packages/ggplot2/index.html). The ComplexHeatmap package (https://www.bioconductor.org/packages/release/bioc/html/ComplexHeatmap.html) was used for heatmap visualization with hierarchical clustering based on Euclidean distance.

## 5. Conclusions

In conclusion, the findings of our study demonstrate for the first time that bovine FF and FF EVs can induce changes in the gene expression of the bovine oviductal cells which, although observed in vitro, may be reflective of in vivo responses, which may contribute to a favorable microenvironment for sperm survival, fertilization, and early embryo development. Although our findings are suggestive of these possible functional roles of follicular fluid, there is a need for more targeted experiments to test whether the transcriptional responses to FF and/or FF-derived EVs translate to measurable phenotypic aspects of reproductive function. Ultimately, our findings highlight a need for further in-depth study of FF and its composition in relation to its possible functional benefits in relation to reproduction and fertility.

## Figures and Tables

**Table 1:** KEGG pathway enrichment analysis results. Genes significantly downregulated (false discovery rate, FDR ≤ 0.05) in both 6 h and 24 h follicular fluid supplementation groups were included in the analysis. Column “differentially expressed (DE Genes)” lists the number of differentially expressed genes at FDR ≤ 0.05 that belong to the corresponding pathway. Organ- or disease-specific pathways with no relevance to the study system were excluded *.

Pathway ID	Pathway Name	DE Genes	FDR
bta00590	Arachidonic acid metabolism	5	0.012
bta00140	Steroid hormone biosynthesis	4	0.026
bta04913	Ovarian steroidogenesis	4	0.026
bta04137	Mitophagy—animal	6	0.026
bta04145	Phagosome	8	0.026
bta04140	Autophagy—animal	8	0.033

* Full list of significantly enriched pathways can be found in Supplementary Table S5.

## Supplementary Materials

The following data are provided online at https://www.mdpi.com/1422-0067/21/15/5365/s1. Table S1. Gene Set Enrichment Analysis (GSEA) results based on differential expression testing results of FF-supplemented BOECs compared to control BOECs at 6 h. Pathways up to FDR ≤ 0.1 are presented. NES-Normalized Enrichment Score. Table S2. Gene Set Enrichment Analysis (GSEA) results based on differential expression testing results of FF-supplemented BOECs compared to control BOECs at 24 h. Pathways up to FDR ≤ 0.05 are presented. NES-Normalized Enrichment Score. Table S3. Gene Set Enrichment Analysis (GSEA) results based on differential expression testing results of EV-supplemented BOECs compared to control BOECs at 6 h. Pathways up to FDR ≤ 0.1 are presented. NES-Normalized Enrichment Score. Table S4. Gene Set Enrichment Analysis (GSEA) results based on differential expression testing results of EV-supplemented BOECs compared to control BOECs at 24 h. Pathways up to FDR ≤ 0.1 are presented. NES-Normalized Enrichment Score. Table S5. Results of pathway over-representation test based on the genes downregulated at both 6 h and 24 h timepoints after FF supplementation. Pathways up to FDR ≤ 0.1 are presented. Supplementary data 1.DE gene list of 6 h FF treatment. Supplementary data 2.DE gene list of 24 h FF treatment. Supplementary data 3.DE genes list of 6 h EV treatment. Supplementary data 4.DE overlap gene list in 6 h with FF and EVs treatment. Supplementary data 5. Overlap upregulated genes with FF treatment in both 6 and 24 h. Supplementary data 6. Overlap downregulated genes with FF treatment in 6 and 24 h.

## Abbreviations

FF	Follicular fluid
EV	Extracellular vesicle
BOECs	Bovine oviductal epithelial cells
DEGs	Differentially expressed genes
GSEA	Gene Set Enrichment Analysis
SEC	size-exclusion chromatography
NTA	Nanoparticle tracking analysis
TEM	Transmission Electron microscopy
WB	Western blot analysis
RNA seq	RNA sequencing
