# Supplementary material for: Bovine Follicular Fluid and Extracellular Vesicles Derived from Follicular Fluid Alter the Bovine Oviductal Epithelial Cells Transcriptome
Source: Int J Mol Sci. 2020 Jul 28;21(15):5365. doi: 10.3390/ijms21155365 (PMC7432463; doi:10.3390/ijms21155365)
Supplement: Supplementary file 1 [file ijms-21-05365-s001.zip › supplementary file/Table S1_S 5.docx]

Table S1. Gene Set Enrichment Analysis (GSEA) results based on differential expression testing results of FF-supplemented BOECs compared to control BOECs at 6h. Pathways up to FDR ≤ 0.1 are presented. NES – Normalized Enrichment Score.

| Pathway ID | Pathway description | NES | FDR |
| --- | --- | --- | --- |
| bta03010 | Ribosome | -1.86 | 0.040 |
| bta03008 | Ribosome biogenesis in eukaryotes | 1.94 | 0.040 |
| bta03013 | RNA transport | 1.64 | 0.090 |
| bta05204 | Chemical carcinogenesis | -1.87 | 0.097 |
| bta00380 | Tryptophan metabolism | -1.80 | 0.097 |
| bta00590 | Arachidonic acid metabolism | -1.81 | 0.097 |
| bta04913 | Ovarian steroidogenesis | -1.79 | 0.097 |
| bta05034 | Alcoholism | -1.81 | 0.097 |
| bta00140 | Steroid hormone biosynthesis | -1.75 | 0.097 |
| bta00830 | Retinol metabolism | -1.73 | 0.097 |

Table S2. Gene Set Enrichment Analysis (GSEA) results based on differential expression testing results of FF-supplemented BOECs compared to control BOECs at 24h. Pathways up to FDR ≤ 0.05 are presented. NES – Normalized Enrichment Score.

| Pathway ID | Pathway description | NES | FDR |
| --- | --- | --- | --- |
| bta05205 | Proteoglycans in cancer | -1.82 | 0.009 |
| bta04510 | Focal adhesion | -1.87 | 0.009 |
| bta04014 | Ras signaling pathway | -1.83 | 0.009 |
| bta03008 | Ribosome biogenesis in eukaryotes | 1.97 | 0.009 |
| bta03040 | Spliceosome | 1.87 | 0.009 |
| bta05012 | Parkinson disease | 1.84 | 0.009 |
| bta03010 | Ribosome | 1.91 | 0.009 |
| bta03013 | RNA transport | 1.97 | 0.009 |
| bta04714 | Thermogenesis | 1.90 | 0.009 |
| bta04810 | Regulation of actin cytoskeleton | -1.77 | 0.010 |
| bta05204 | Chemical carcinogenesis | -1.83 | 0.010 |
| bta03050 | Proteasome | 1.84 | 0.010 |
| bta04110 | Cell cycle | 1.77 | 0.010 |
| bta00190 | Oxidative phosphorylation | 1.78 | 0.010 |
| bta05016 | Huntington disease | 1.69 | 0.010 |
| bta04137 | Mitophagy - animal | -1.76 | 0.014 |
| bta04144 | Endocytosis | -1.59 | 0.015 |
| bta05206 | MicroRNAs in cancer | -1.66 | 0.023 |
| bta04520 | Adherens junction | -1.71 | 0.023 |
| bta00230 | Purine metabolism | 1.71 | 0.024 |
| bta04140 | Autophagy - animal | -1.66 | 0.024 |
| bta04142 | Lysosome | -1.69 | 0.024 |
| bta00980 | Metabolism of xenobiotics by cytochrome P450 | -1.73 | 0.025 |
| bta04650 | Natural killer cell mediated cytotoxicity | -1.72 | 0.026 |
| bta04932 | Non-alcoholic fatty liver disease (NAFLD) | 1.63 | 0.026 |
| bta05211 | Renal cell carcinoma | -1.73 | 0.027 |
| bta00380 | Tryptophan metabolism | -1.71 | 0.027 |
| bta04015 | Rap1 signaling pathway | -1.62 | 0.028 |
| bta04370 | VEGF signaling pathway | -1.70 | 0.034 |
| bta05200 | Pathways in cancer | -1.41 | 0.034 |
| bta04723 | Retrograde endocannabinoid signaling | 1.60 | 0.034 |
| bta03460 | Fanconi anemia pathway | 1.67 | 0.040 |
| bta05165 | Human papillomavirus infection | -1.46 | 0.041 |
| bta05223 | Non-small cell lung cancer | -1.67 | 0.042 |
| bta04360 | Axon guidance | -1.59 | 0.0423 |
| bta04915 | Estrogen signaling pathway | -1.64 | 0.043 |
| bta04512 | ECM-receptor interaction | -1.67 | 0.049 |
| bta00270 | Cysteine and methionine metabolism | 1.65 | 0.049 |
| bta05218 | Melanoma | -1.65 | 0.049 |
| bta03020 | RNA polymerase | 1.66 | 0.049 |

Table S3. Gene Set Enrichment Analysis (GSEA) results based on differential expression testing results of EV-supplemented BOECs compared to control BOECs at 6h. Pathways up to FDR ≤ 0.1 are presented. NES – Normalized Enrichment Score.

| Pathway ID | Pathway description | NES | FDR |
| --- | --- | --- | --- |
| bta04530 | Tight junction | 1.56 | 0.052 |
| bta05220 | Chronic myeloid leukemia | 1.61 | 0.052 |
| bta05204 | Chemical carcinogenesis | -1.84 | 0.079 |
| bta04510 | Focal adhesion | 1.49 | 0.079 |
| bta05221 | Acute myeloid leukemia | 1.58 | 0.079 |
| bta05206 | MicroRNAs in cancer | 1.49 | 0.079 |
| bta00190 | Oxidative phosphorylation | -1.81 | 0.079 |
| bta05012 | Parkinson disease | -1.92 | 0.079 |
| bta03010 | Ribosome | -2.43 | 0.079 |
| bta00983 | Drug metabolism - other enzymes | -1.76 | 0.079 |
| bta05010 | Alzheimer disease | -1.62 | 0.079 |
| bta04390 | Hippo signaling pathway | 1.5 | 0.079 |
| bta04723 | Retrograde endocannabinoid signaling | -1.65 | 0.079 |
| bta05135 | Yersinia infection | 1.51 | 0.079 |
| bta05016 | Huntington disease | -1.53 | 0.079 |
| bta00513 | Various types of N-glycan biosynthesis | 1.57 | 0.083 |
| bta05226 | Gastric cancer | 1.52 | 0.087 |
| bta04714 | Thermogenesis | -1.36 | 0.087 |
| bta05216 | Thyroid cancer | 1.56 | 0.087 |
| bta04713 | Circadian entrainment | -1.64 | 0.087 |
| bta04010 | MAPK signaling pathway | 1.4 | 0.087 |
| bta05223 | Non-small cell lung cancer | 1.53 | 0.087 |
| bta05212 | Pancreatic cancer | 1.51 | 0.087 |
| bta04612 | Antigen processing and presentation | -1.59 | 0.093 |
| bta05031 | Amphetamine addiction | -1.62 | 0.093 |
| bta04740 | Olfactory transduction | -1.63 | 0.097 |
| bta04920 | Adipocytokine signaling pathway | 1.52 | 0.097 |

Table S4. Gene Set Enrichment Analysis (GSEA) results based on differential expression testing results of EV-supplemented BOECs compared to control BOECs at 24h. Pathways up to FDR ≤ 0.1 are presented. NES – Normalized Enrichment Score.

| Pathway ID | Pathway name | Normalized enrichment scores (NES) | FDR |
| --- | --- | --- | --- |
| bta00190 | Oxidative phosphorylation | 1.6788 | 0.0448 |
| bta03010 | Ribosome | 1.9935 | 0.0448 |
| bta05016 | Huntington disease | 1.59315 | 0.04485 |
| bta05332 | Graft-versus-host disease | 1.8181 | 0.0448 |
| bta05012 | Parkinson disease | 1.5743 | 0.0448 |
| bta04640 | Hematopoietic cell lineage | 1.8464 | 0.0448 |
| bta04260 | Cardiac muscle contraction | 1.8248 | 0.0529 |
| bta05010 | Alzheimer disease | 1.5457 | 0.0533 |
| bta04940 | Type I diabetes mellitus | 1.7912 | 0.0645 |
| bta03008 | Ribosome biogenesis in eukaryotes | -1.5956 | 0.0857 |
| bta04066 | HIF-1 signaling pathway | 1.5973 | 0.0971 |
| bta04062 | Chemokine signaling pathway | 1.5480 | 0.0971 |
| bta05321 | Inflammatory bowel disease (IBD) | 1.7238 | 0.0971 |
| bta05320 | Autoimmune thyroid disease | 1.7209 | 0.0971 |
| bta05330 | Allograft rejection | 1.7375 | 0.0971 |

Table S5. Results of pathway over-representation test based on the genes downregulated at both 6h and 24h timepoints after FF supplementation. Pathways up to FDR ≤ 0.1 are presented.

| Pathway ID | Pathway description | Gene ratio | Background ratio | FDR |
| --- | --- | --- | --- | --- |
| bta00590 | Arachidonic acid metabolism | 5/82 | 22/4047 | 0.0123 |
| bta04966 | Collecting duct acid secretion | 4/82 | 16/4047 | 0.0240 |
| bta00140 | Steroid hormone biosynthesis | 4/82 | 21/4047 | 0.0255 |
| bta04913 | Ovarian steroidogenesis | 4/82 | 21/4047 | 0.0255 |
| bta04137 | Mitophagy - animal | 6/82 | 55/4047 | 0.0255 |
| bta05323 | Rheumatoid arthritis | 6/82 | 55/4047 | 0.0255 |
| bta04145 | Phagosome | 8/82 | 101/4047 | 0.0256 |
| bta04140 | Autophagy - animal | 8/82 | 107/4047 | 0.0327 |
| bta04672 | Intestinal immune network for IgA production | 3/82 | 15/4047 | 0.0690 |
